# The Features of Martensitic Transformation in 12% Chromium Ferritic–Martensitic Steels

**DOI:** 10.3390/ma14164503

**Published:** 2021-08-11

**Authors:** Kseniya Bazaleeva, Alexander Golubnichiy, Anton Chernov, Andrey Ni, Ruslan Mendagaliyev

**Affiliations:** 1 Institute of Innovative Engineering Technologies, RUDN University, 117198 Moscow, Russia; ni-av@rudn.ru; 2 Institute of Manufacturing Technologies and Engineering, Moscow State University of Technology «STANKIN», 109240 Moscow, Russia; 3 Scientific Research, Design and Technological Department of Development of Fuel Rods for Fast and Gas Reactors, JSC A. A. Bochvar High-Technology Research Institute of Inorganic Materials, 123098 Moscow, Russia; sbux27@yandex.ru (A.G.); chernoff95@gmail.com (A.C.); 4 World-Class Research Center «Advanced Digital Technologies», St. Petersburg State Marine Technical R, 190121 St. Petersburg, Russia; ruslanm888@mail.ru

**Keywords:** martensitic transformation, thermal dilatometric analysis, ferritic–martensitic steels, microstructure

## Abstract

An anomaly in martensitic transformation (the effect of martensitic two-peak splitting in the temperature-dependent thermal expansion coefficient) in complex alloyed 12% chromium steels Fe-12%Cr-Ni-Mo-W-Nb-V-B (ChS-139), Fe-12%Cr-Mo-W-Si-Nb-V (EP-823) and Fe-12%Cr-2%W-V-Ta-B (EK-181) was investigated in this study. This effect is manifested in steels with a higher degree of alloying (ChS-139). During varying temperature regimes in dilatometric analysis, it was found that the splitting of the martensitic peak was associated with the superposition of two martensitic transformations of austenite depleted and enriched with alloying elements. The anomaly was subsequently eliminated by homogenization of the steel composition due to high-temperature aging in the γ-region. It was shown that if steel is heated to 900 °C, which lies in the (α + γ) phase region or slightly higher during cooling, then the decomposition of austenite proceeds in two stages: during the first stage, austenite is diffused into ferrite with carbides; during the second stage, shear transformation of austenite to martensite occurs.

## 1. Introduction

When highly alloyed chromium steels are considered as prospective construction materials for active zones in fusion reactors and nuclear fast neutron reactors [1,2,3,4,5,6,7,8,9,10], thermal stability is one of the aspects determining their application [11,12,13]. The structure and properties of ferritic–martensitic steels are formed as a result of heat treatment, which usually consists of air quenching at 1080–1100 °C with subsequent tempering at 680–720 °C [14,15]. However, variations in heat treatment conditions in these multicomponent systems, namely, changes in the quenching temperature, exposure time, cooling rate, and tempering time, lead to the redistribution of carbide-forming elements and carbon between solid solutions and carbide phases, which affects the structure and structural stability of steel [16].

During thermal analysis of ferritic–martensitic steel of the Fe-12%Cr-Ni-Mo-W-Nb-V-B (ChS-139) system, an anomaly in martensitic transformation was detected in a study [17] using a dilatometric method. The process of cooling from a high-temperature γ-area at a speed of 15 K/min to the temperature range of 200–300 °C is when the only γ→α_m_ phase transformation occurs in steel (where α_m_ is martensite), which in the diagram of the thermal expansion, physical coefficient α should correspond to one peak. However, it was observed that there were two crossed peaks (splitting of the martensitic peak) on the experimental cooling curve in this temperature range. A thermal analysis of steel of the same composition was also carried out in [15,18,19]; however, during the cooling of the steel from the γ-phase region, in addition to the martensitic transformation, no other effects were recorded. The reason for the anomaly in the martensitic transformation observed in [17], which may be the result of the superposition of two structural transformations, was not identified in the study, but this reason is likely to affect the structural state formed as a result of heat treatment. From this point of view, it seems relevant to establish the effect of the splitting of the martensitic peak on the temperature dependencies of the α coefficient upon cooling of 12% chromium steels of various alloying systems, and to identify the cause of its occurrence.

## 2. Materials and Methods

The following ferritic–martensitic steels were chosen as the objects of study: Fe-12%Cr-2%W-V-Ta-B (EK-181), Fe-12%Cr-Mo-W-Si-Nb-V (EP-823), and Fe-12%Cr-Ni-Mo-W-Nb-V-B (ChS-139). The chemical content is given in Table 1.

**Figure 1:**
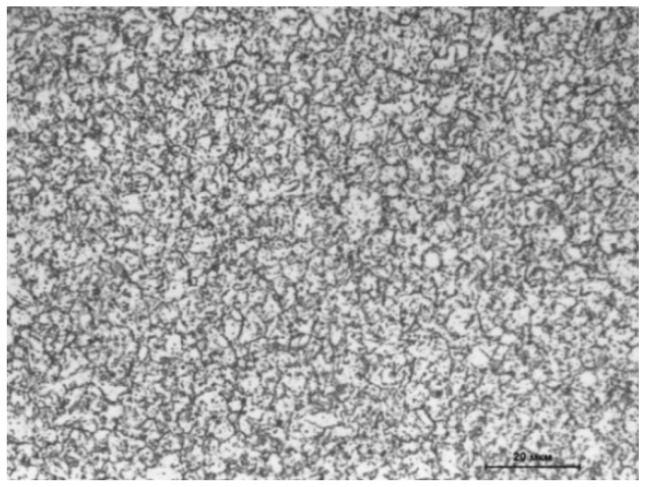
Structure of the ChS-139 steel after annealing.

The initial structure of the steels under study corresponded to the annealed condition (T_ann_ = 780 °C, τ_ann_ = 10 h, cooling in furnace down to 600 °C, and further cooling in open air) attaining a ferritic–carbide state (Figure 1). Samples for dilatometric analysis were prepared on a turner, attaining a cylindrical shape with a diameter and length of 6 and 25 mm, respectively.

The structures obtained after dilatometric analysis were studied using metallographic and X-ray diffraction methods, as well as scanning electron microscopy. Most metallographic specimens were prepared by electrolytic etching in an aqueous solution of 3% oxalic acid at a voltage of 8 V and a current density of 0.9 mA/cm^2^ for 50 s. However, the etching of samples that were heated to temperatures ranging from 825 to 900 °C during dilatometric analysis was carried out chemically in the solution (50 mL of HF + 20 mL of H_2_SO_4_ + 1500 mL of H_2_O). The surfaces of the samples for X-ray diffraction analysis were prepared by electrolytic polishing in the solution (600 mL methanol + 360 mL butyl glycol + 60 mL 60% HClO_4_) at a voltage of 20 V and a current density of 0.5 mA/cm^2^ for 20 s. The samples for scanning electron microscopy, after mechanical grinding, were polished using a diamond suspension with a fineness of 1 μm.

Metallographic analysis of the obtained structures was carried out using an Olympus GX51 optical microscope (Olympus, Tokyo, Japan), and scanning electron microscopy was performed using a Carl Zeiss EVO LS 15 microscope (Carl Zeiss, Jena, Germany). Microhardness values were determined on a DuraScan-80 microhardness tester (Emco-Test, Kuchl, Austria) at a load of 50 g with a relative error of 5%.

The dilatometric analysis was conducted using a DIL 402C (Netzsch, Selb, Germany) with alundum equipment (Netzsch, Selb, Germany). The heating and cooling of samples were applied at a speed of 15 K/min. The samples of EK-181 and EP-823 steels were heated to 1200 °C and immediately cooled at the same speed. The heat temperature of the steel ChS-139 was varied from750 to1350 °C. There were three regimes for measurements: without exposure at the high-temperature γ-range, and with exposure for 1 h and 3 h. To prevent surface oxidization, the chamber was ventilated by argon at a rate of 100 mL/min. The temperature in the chamber was measured using a tungsten–rhenium thermocouple (Netzsch, Selb, Germany) W3%Re/W25%Re type B. The error of temperature measurement was in the 3° range. Correction curves were plotted for every experimental regime using alundum cylindrical samples of the same size as the experimental samples. According to the measurements of the sample’s length L depending on the temperature T, for each T value, the relative change of length δ was determined as

δ = (L_2_ − L_1_)/L1 × 100% (1)

and the α value as

α = (dL/dT) × (1/L)(2)

The heating and cooling of the alloy were accompanied by a monotonous change in linear dimensions according to α failing the phase transformation. However, the phase transition was accompanied by a drastic change in the linear sample’s dimensions. Thus, temperature dependence α(T) shows a peak on the plot. It is important to note that for each steel type, dilatometric analysis was carried out on 5 samples.

The phase structure determination was conducted using a D8 Advance X-ray diffractometer (Bruker, Rheinstetten, Germany) with Co Kα irradiation at a diffraction angle range of 2ϑ = 40–130 degrees with a 0.07 degree step and exposition of 1.5 s at a point. The slit system was as follows: the motorized slit in the tube and the second slit in the counter measured 1 and 5 mm, respectively, and Soller’s slits within the distance between plates measured 2.5 mm. The quantitative phase analysis was carried out using Diffrac.Eva software (version Diffrac.Eva V5.1, Bruker, Rheinstetten, Germany) with a relative phase analysis error of 20%.

## 3. Results

### 3.1. The Martensitic Peak Splitting Effect on Steels of Different Content

**Figure 2:**
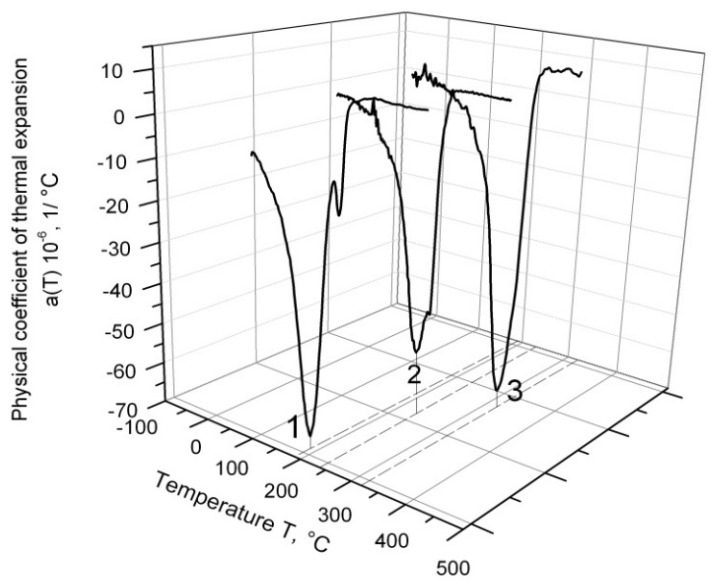
Temperature dependence of thermal expansion coefficient for ChS-139 (1), EP-823 (2), and EK-181 steels (3).

The temperature dependence of α for different ferritic–martensitic alloys cooling down from 1200 °C is depicted in Figure 2. The effect of martensitic peak splitting was absent in all samples of EK-181 steel (line 3), and only two samples of EP-823 steel displayed this effect (line 2); however, it was more strongly presented in all samples of ChS-139 steel (line 1). Therefore, further investigation was carried out only on ChS-139.

In Figure 2, it can be observed that in the series of EK-181, EP-823, and ChS-139 alloys, the temperature of martensitic transformation shifted to the low-temperature region.

### 3.2. The Influence of High-Temperature Exposure on the Martensitic Peak Split Effect

**Figure 3:**
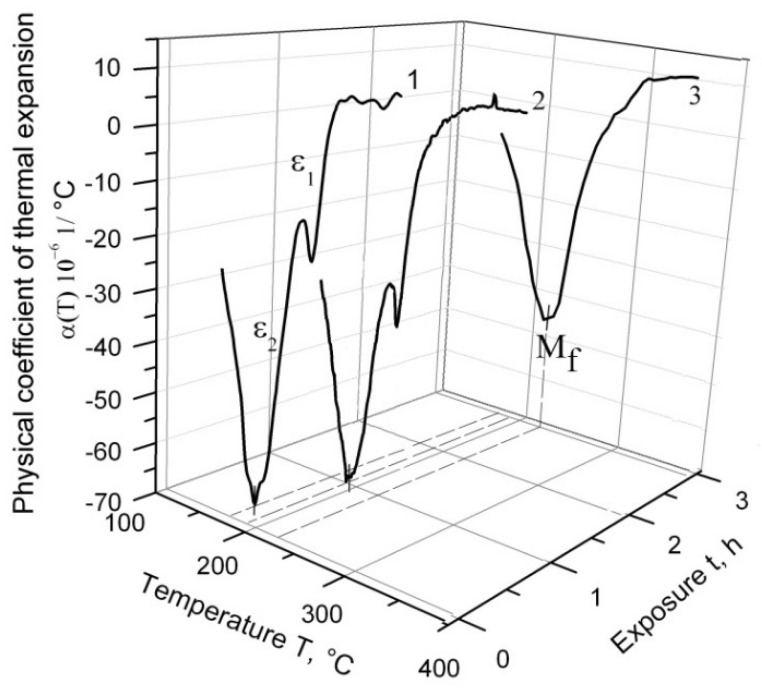
The influence of exposure at the high-heat region on the effect of martensitic peak splitting: 1—without exposure, 2—1 h exposure, and 3—3 h.

Figure 3 depicts the temperature dependence of α(T) during the cooling process of ChS-139 steel from 1200 °C following three temperature regimes: with 1 h exposure at a high temperature range, with 3 h exposure, and without exposure. According to the obtained results, the splitting effect of the martensitic peak into ε_1_ and ε_2_ was observed on the curve of α(T) cooling without exposure, and the main fraction of austenite underwent transformation at ε_2_. The exposure of 1 h in the γ-region caused a decrease in ε_1_. Without exposure, this effect corresponded to the relative change in the sample length, δ ≈ 0:007%, and after exposure of 1 h, to δ ≈ 0:003%. After 3 h exposure at 1200 °C, the splitting effect disappeared.

In Figure 3, it can be observed (at exposure in the high-temperature region) that at the end of martensitic transformation, ε_2_ shifted to the higher temperature values range (M_f_ point).

### 3.3. The Influence of Heat Temperature on the Martensitic Peak Split Effect

**Figure 4:**
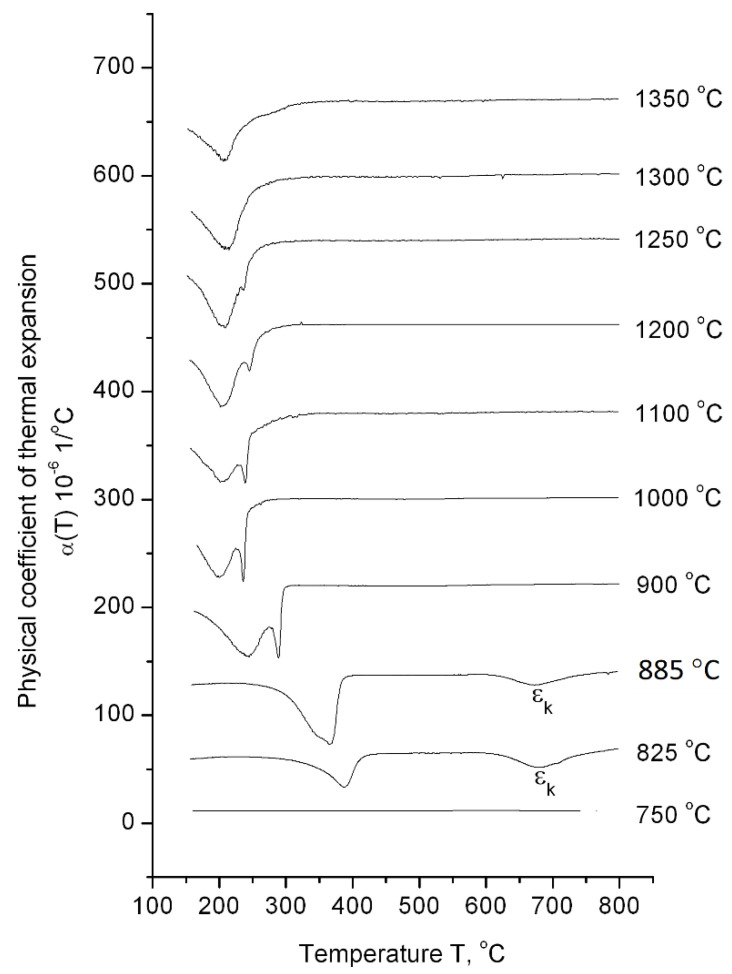
The effect of heating temperature on martensitic peak splitting.

The dilatometric analysis results of ChS-139 steel cooling from 1350 to 750 °C after exposure for 1 h in the high-temperature region is presented in Figure 4. According to the dilatometric heating curve, the temperature interval of α→γ transformation was determined as 820–850 °C; i.e., the 750 °C value corresponds to the area of low-temperature α-ferrite existence, the 825 °C − (α + γ)-phase area, and the interval 885–1350 °C corresponds to austenite existence. At the temperature value of 750 °C, neither during heating process nor during cooling down any phase transformation was detected in the dependence curves of α(T). This indicates that the steel preserves the initial ferrite–carbide state over the entire temperature range (from room temperature to 750 °C). In this structural state, the steel has a microhardness value of 250 HV_0.05_ (Table 2).

At heating temperatures of 900–1350 °C, the only γ→α_m_ transformations were observed in the 200–300 °C range of the cooling curves. Moreover, at 900–1250 °C, the transformation was described by a split peak, and at higher temperatures, the splitting effect disappeared. The maximum value of ε_1_ effect was observed at a heating temperature of 1000 °C (the relative change in the sample length for this regime was 0.016%). As heating temperature increased, the relative change in the length of the sample during ε_1_ transformation decreased, amounting to 0.001% at the heating temperature of 1250 °C.

At the heating temperatures of 825 °C and 885 °C, corresponding to the two-phase (γ + α) range and interface between (γ + α) and γ, respectively, the splitting effect of the martensitic peak was not observed. Moreover, additional ε_k_ transformation was located in the temperature range of 680–750 °C.

It is important to note that starting from 900 °C, the temperature decrease initiated a shift of the martensitic points toward higher temperatures.

### 3.4. The Impact of Heating Temperature onSteel Structure

**Figure 5:**
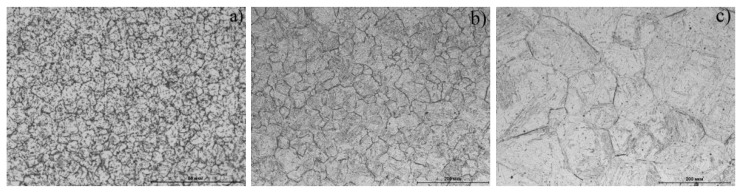
The microstructures of ChS-139 steel after cooling down from (**a**) 825 °C, (**b**) 1200 °C, and (**c**) 1300 °C.

The steel structures were studied using metallography analysis (Figure 5) and microhardness measurement (Table 2) after cooling down from various temperatures. The martensitic structure with a hardness of ≥600 HV_0.05_ with an array of dark formations on the initial austenitic grain boundaries was formed at a heating range of 1000–1350 °C (Figure 5b,c). The dark formations were presumably carbide inclusions. The increase in temperature in this range led to an increase in the initial γ-phase grain and to a decrease in formation concentration along the boundaries due to dissolution.

A fundamentally different structure was formed when the steel was heated into a two-phase (γ + α) area (825 °C) and slightly higher 885 °C (Figure 5a). Despite the presence of a peak on the dilatometric curve that corresponded to martensitic transformation, the observed structure was a ferrite–carbide mixture; the microhardness value corresponded to the annealed structure (Table 2).

**Figure 6:**
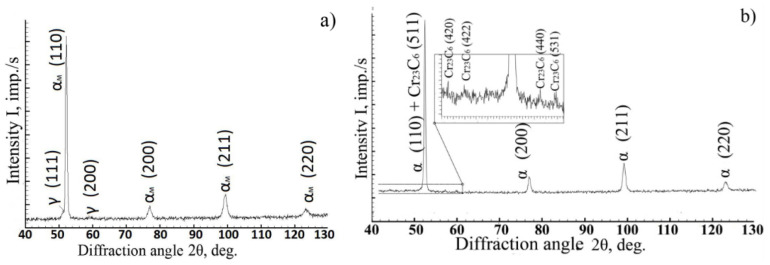
Diffraction patterns of ChS-139 steel after cooling down from 900 °C (**a**) and 885 °C (**b**).

Using X-ray phase analysis, it was found that the diffraction patterns of steel cooled down from 900–1350 °C contained reflections from the martensite crystal lattice and intense reflections of residual austenite (Figure 6a). The amount of residual austenite was 4–5%, while the amount of carbide phases in these structures was below the sensitivity limit of the method. Upon cooling from lower temperatures, reflections from the α-phase and the Me_23_C_6_-type carbide were recorded in the diffraction pattern. Moreover, the diffraction maxima of the α-phase were much narrower than they were upon cooling from higher temperatures (Figure 6b).

**Figure 7:**
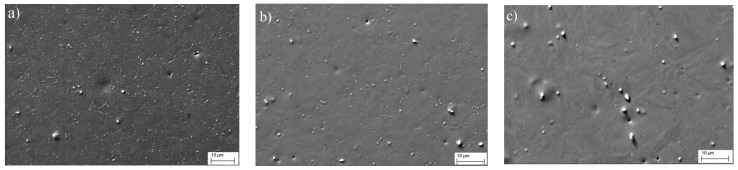
The influence of heating temperature on the distribution of carbide phases in the structure of ChS-139 steel (SEM): heating temperature of (**a**) 885 °C; (**b**) −900 °C; (**c**) −1200 °C.

Figure 7 shows the microstructures of steel cooled down from different phase regions (SEM). Shown contrast due to back-scattered and secondary electrons, the light-colored particles represent the carbides. It can be observed from the figure that while maintaining the average concentration of large micron-sized particles, apparently of metallurgical origin, the quantity of small secondary carbides increased noticeably with a decrease in the steel heating temperature; i.e., the process of diffusion decomposition proceeded much more actively during the cooling of the steel from the temperature region of polymorphic α⇄γ transformation.

## 4. Discussion

### 4.1. The Nature of the Martensitic Peak Splitting Effect

It can be assumed that the martensitic peak splitting effect was associated with the level of steel alloying; i.e., the decreasing temperature in martensitic transformation increased the tendency of alloy to the splitting effect of the martensitic peak in the sequence of EK-181, EP-823, and ChS-139, as evidenced by the gain in the degree of alloying. It is likely that in the steels with a higher concentration of alloying elements, where diffusion processes were substantially slowed down, the composition of the austenitic solid solution before quenching/cooling was inhomogeneous. Since austenite composition affects the temperature value onset of M_s_ and the end of M_f_ in martensitic transformation, the inhomogeneity of the γ-phase should lead to the appearance of an expanded martensitic peak on the graph of α(T). This expanded martensitic peak is a superposition set of individual peaks, where each peak corresponds to the γ→α_m_ transformation of austenite of a certain composition. However, this experiment observed a splitting into two peaks: ε_1_ and ε_2_, as shown in Figure 3. It is possible that the inhomogeneity of the austenitic solid solution occurred during the heating of the steel into a single-phase γ region through a two-phase (α + γ). With the passage of the steel structure through the two-phase region, an enriched with alloying elements ferrite and a depleted austenite appeared. The leveling of the austenite composition continues after transition to the single-phase region, where the γ-solid solution of the two compositions (enriched and depleted with alloying elements) was subjected to cooling. If the exposure time were increased in the single-phase region or the heating temperature, the diffusion processes would run more completely. Therefore, the latter should lead to the complete or partial disappearance of the martensitic peak splitting effect. To verify this assumption, a dilatometric analysis of steel was carried out using various modes, namely, heating to constant temperature (1200 °C), but with different regimes: without exposure, with 1 h and 3 h exposures, and with heating to different temperatures with the same exposure time of 1 h.

### 4.2. The Effect of Homogenization of γ-Solid Solution on Martensitic Peak Splitting

Assuming that peak ε_1_ corresponded to the transformation of depleted with alloying elements austenite into martensite, which fraction was small, and peak ε_2_ referred to the transformation of enriched with alloying elements austenite, then the compositions of depleted and enriched γ-solid solutions would be expected to approach each other during high-temperature aging. In this case, the temperature ranges of the transformations ε_1_ and ε_2_ would also shift towards each other along the temperature scale until they completely coincided. For peak ε_2_, which corresponded to the conversion of the main part of austenite, this effect was established experimentally with an increase in the exposure time from 0 to 3 h at heating temperature of 1200 °C (Figure 3). An increase in the heating temperature, which also promoted the homogenization of the γ-solid solution, led to the gradual disappearance of the ε_1_ peak. As shown in Figure 4, the martensitic peak splitting was at its maximum at 1000 °C. With increasing temperature, this effect decreased, disappearing completely at 1300 °C, since at higher temperatures, there was more time for the homogenization of the austenitic solid solution.

Thus, dilatometric studies carried out under different conditions showed that after prolonged exposure (3 h) in the austenitic region and upon heating to (≥1300 °C), the cooling of the steel was not accompanied by the splitting of the martensitic peak; i.e., the effect disappeared with homogenization of the solid solution. Undoubtedly, the study showed that the level of homogeneity of the martensite formed during cooling affects the structure and properties of steel after tempering.

### 4.3. Diffusion Transformations during Cooling

It is known that in ferritic–martensitic chromium steels, the decomposition of austenite during cooling can run two ways—partially according to the diffusion stage with the formation of a ferrite–carbide mixture and partially shear to the formation of martensite [16]. It is likely that the ε_k_ effect observed upon the cooling of steel from temperatures of 825 and 885 °C (Figure 4) is related to the diffusion stage of austenite decomposition. The shift in the temperature range for the martensitic transformation of steel to the higher values up to T ≤ 900 °C was also a consequence of the presence of the diffusion stage of austenite decomposition in the temperature range of 680–750 °C. At this stage, the austenite was depleted in carbon and alloying elements, which led to a shift in the temperatures of martensitic transformation towards higher values. This was due to the speed of the diffusion processes. The total concentration of alloying elements in ChS-139 steel is greater than in EP-823 steel. Thus, we can say that the diffusion processes in ChS-139 steel proceed slower.

The appearance of the ChS-139 steel ferrite–carbide structure after cooling down from 825–885 °C was unexpected. The γ→α_m_ transformation under these regimes occurred at ~400 °C. It may be assumed that the high temperature of the martensitic transformation and the presence of diffusion decay products of austenite in the structure caused tempering in the longer cooling period.

The assumption made on the basis of the results in the thermal analysis on the diffusion nature of the transformations observed upon cooling of steel from temperatures below 900 °C was confirmed by X-ray detection of the Me_23_C_6_ carbide phase in the diffraction patterns of steel after heating to 825 and 885 °C (Figure 6) and by electron microscopic studies of the distribution of carbide particles in the steel (Figure 7), which indicated an increase in the number of dispersed carbide inclusions with decreasing heating temperature.

## 5. Conclusions

Using the dilatometric analysis method, it was shown that upon cooling from the high-temperature region of Fe-12%Cr-Ni-Mo-W-Nb-V-B (ChS-139) steel, the thermal effect of the martensitic transformation had a bifurcated nature: there was an overlap of two martensitic transformations of enriched and depleted with alloying elements austenite. This observation was much less noticeable in Fe-12%Cr-Mo-W-Si-Nb-V (EP-823) steel, and in Fe-12%Cr-2%W-V-Ta-B (EK-181), it was absent.

The variation in the dilatometric analysis mode of ChS-139 steel, namely, an increase in the exposure time and heating temperature, made it possible to establish that the effect of the splitting of the martensitic peak can be eliminated by homogenization of the austenitic solid solution.

At temperatures below 900 °C in ChS-139 steel, the decomposition of austenite proceeded in two stages: at a temperature of 680–750 °C, a diffusion stage was observed, and at temperatures of ~400 °C, martensitic transformation occurred. After such heating conditions, martensite was not observed in the structure. Only a ferrite–carbide mixture with a hardness corresponding to the annealed state of steel was present. It is likely that a sufficiently high temperature of martensitic transformation and the presence of diffusion decay products enabled the release of martensite during cooling.

## Figures and Tables

**Table 1:** Chemical content of experimental steels, mass % *.

Steel	C	Cr	Ni	W	Mo	Nb	V	Ta	Ti
EK-181	0.12–0.18	10–12	≤0.05	0.8–1.5	≤0.05	≤0.03	0.2–0.4	≤0.1	0.01
EP-823	0.14–0.18	10–12	0.5–0.8	0.5–0.8	0.6–0.9	0.2–0.4	0.2–0.4	0.15	0.01
ChS-139	0.19–0.20	11–12.5	0.5–0.8	1–1.5	0.4–0.6	0.2–0.4	0.2–0.4	0.07–0.15	0.01

* The alloy base is Fe.

**Table 2:** Dependence of the ChS-139 steel microhardness on heating temperature.

Temperature °C	Init.	750	825	885	900	1000	1100	1200	1250	1300	1350
HV_0.05_	252	250	325	262	545	600	637	615	613	634	642

## Data Availability

Not applicable.
